# Progress in Surgery

**Published:** 1903-01-03

**Authors:** 


### Progress in Surgery.

### OTOLOGY.

**Suppurative Otitis Media.**—C. H. Fagge[1] has published remarks on this affection. Statistics as to the frequency of ear suppuration in childhood were stated to be unattainable. But Cheatle has reported that of 1,000 Board-school children examined in the Hanwell schools, 335 were suffering, or had suffered, from this affection. The writer then quotes Pitt's statistics to show that fatal complications from this affection are not mythical. Pitt showed that out of 9,000 autopsies, suppurative otitis was the known cause of death in 1 in 158, and in addition probably was the cause of other deaths attributed to pyæmia, convulsions, and other diseases. Complications were most common between the ages of 11 and 30, between which years 58 per cent. of all complications occurred, and, according to Körner, probably those complications form 4·5 per cent. of all causes of death recorded during this period of life. Influenza was the specific fever which most frequently led to troublesome otorrhœa and to intracranial complications. Tubercle as a cause, it was suggested, was most common in extreme infancy, and the disease led to tubercular meningitis in some cases. A diagnostic point in favour of tubercular otitis was a gradual enlargement of the infra-mastoid lymph glands in a child with chronic otorrhœa. Apart from specific fevers, a common cold was the next most frequent cause of chronic otorrhœa. When severe intermittent earache and deafness of sudden onset were met with in childhood, it was of the greatest importance to recognise the injected, opaque, often bulging drum of the acute stage of inflammation in order that suppuration might be prevented. Of the prophylactic measures which could be used, paracentesis was undoubtedly the most important. In the painful stage of acute otitis it was recommended to inject 1 in 40 carbolic lotion, as hot as possible, into the meatus, after first thoroughly cleansing that passage with soap, turpentine, and ether. Thus the way was paved for paracentesis. Fagge thought that if severe pain resisted local medication for 24 hours, and if the redness of the membrane remained, then paracentesis should be performed through the posterior inferior segment of the drum. It would later be found that under local antiseptic treatment and the daily use of Politzer's bag the operation wound healed. It was the writer's experience that bilateral chronic middle-ear suppuration in childhood was in the majority of cases associated with adenoids, and that treatment was unavailing without their removal. If attempts, in acute cases, had failed to prevent suppuration, it was best to cleanse the meatus daily and instil 33 per cent. alcohol drops and pack with gauze. Macleod Yearsley [2] has written a paper on chronic ear discharges. The character of the discharge was usually purulent, and it was rare to find more mucus than pus, and the amount was greatest in the scarlatino-diphtheritic form or when granulation tissue or caries of the temporal bone was present. The most frequent position for perforation was in the antero-inferior portion of the membrane, the next most frequent position was in the postero-superior quadrant, and the third most common was in Schrapnell's membrane. One mode of getting the whistling sound, in cases where Eustachian obstruction interfered with the usual method, was to inflate through the meatus and have the diagnostic tube in the patient's nostril. The perforations in the postero-superior quadrant were often very difficult to treat, being frequently complicated with caries of the incus. Those of Schrapnell's membrane were often associated with caries of the head of the malleus. In both cases there was a liability to suppuration in the mastoid antrum. Cholesteotomata were formed by excessive desquamation of epithelial ingrowth from the external meatus through the perforation in the drumhead. Thus large collections of dead epithelial masses might be formed which were dangerous owing to the intracranial sequelæ they were liable to induce, and further, middle-ear suppuration could not be made to cease until such masses were removed. For the cure of otitis the writer states that it is quite as important to remove adenoids in adults as in children. If vertigo occurs when syringing an ear, it can be relieved by using Politzer's bag or by rarefying the air in the meatus with Siegle's speculum. The air-douche should always be used gently in suppurative cases, lest the antrum be infected, and the danger of this was greater if the suppuration affected the attic region. The antiseptics mentioned for syringing the ear by the meatus were solutions of carbolic acid (1 in 40), lysol (1 to 2 per cent.), resorcin (2 to 3 per cent.), boric acid (ʒj. to a pint), perchloride of mercury (1 in 3,000 to 4,000), or permanganate of potassium. It was useless for the patient to attempt to syringe his own ear. The insufflation of dry powders into the ear was a method which might end in disaster, and fluid instillations were preferable. Peroxide of hydrogen was one of the best fluids for instillation, especially in cases in which there was much inspissated pus, epithelial débris, etc. It should be used before syringing and not previously heated. In the absence of caries and cholesteotomata a useful form of treatment was to purify the meatus and tympanum as thoroughly as possible with 1 in 40 carbolic, dry it, and then swab out with Lister's "strong mixture," which is a solution of 1 in 25 carbolic acid, with 1·500th part of perchloride of mercury. The meatus was then packed with double cyanide gauze soaked in 1 in 40 carbolic and left for 24 to 48 hours. The effect was often astonishing. E. B. Dench (New York) [3] has contributed a paper on various operations for the relief of chronic suppuration in the ear. In all the persistent and long-standing cases the cause of the discharge was diseased bone within the tympanic cavity. The author showed that the simple operation of removal of the ossicles and thorough curettement of the tympanum effected a cure in at least one-half of the cases operated upon, and he advised the operation providing the cases were carefully selected and when the caries was limited to the ossicles, or to the ossicles and those parts of the tympanic cavity which were easily accessible through the external auditory meatus. It was necessary to completely remove the incus, as this ossicle was the most frequent initial seat of this intra-tympanic caries, and even though only a small fragment of the ossicle remained this would be sufficient to keep up the suppuration. The author advised entering the mastoid antrum as the first step in practically every case in which the radical operation was indicated. The outer two-thirds of the posterior meatal wall should be removed completely, *i.e.* from the level of the roof to that of the floor of the meatus. But the inner third of the posterior wall should not be removed so completely, owing to the danger of injuring the facial nerve or the horizontal semicircular canal. To provide a cutaneous lining to the cavities the author had found it advisable to take tissue from the concha as well as from the meatus, and to dissect out the fibro-cartilaginous tissue from these flaps. In many cases after the radical operation the hearing remained the same as before, in others it was made worse, and in others improved. The patient should therefore be warned beforehand that audition might be greatly impaired by the operation. T. R. Chambers [4] advocates the use of hot-water douching to abort mastoiditis. He asserts that if the douche is used for 20 minutes at each seance, the water being at 120° Fahr., it will very often abort purulent otitis and acute mastoiditis, or exacerbations of chronic mastoiditis. The use of the return-current ear douche was absolutely necessary to avoid blistering from the very hot water.

[1] Clin. Journ., May 28, 1902, p. 86. [2] Med. Tim. and Hosp. Gaz., June 28, 1902, p. 401. [3] Laryngoscope, Aug. 1902, p. 624. [4] Med. Rec., Aug. 2, 1902, p. 194.

(*To be continued*)

